# *QuickStats:* Percentage* of Adults Aged ≥18 Years Who Took Medication To Help Fall or Stay Asleep Four or More Times in the Past Week,^†^ by Sex and Age Group — National Health Interview Survey, United States, 2017–2018^§^

**DOI:** 10.15585/mmwr.mm6849a5

**Published:** 2019-12-13

**Figure:**
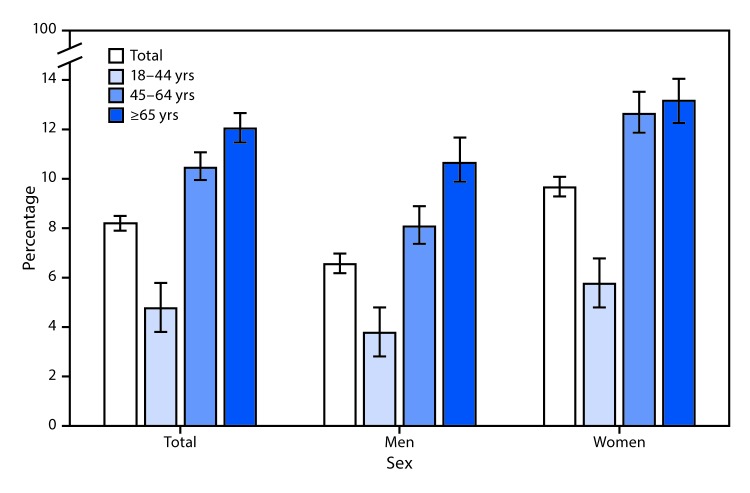
During 2017–2018, 8.2% of adults aged ≥18 years took medication to help fall or stay asleep four or more times in the past week (6.6% for men and 9.7% for women). Among men, the percentage who took medication for sleep four or more times in the past week increased with age from 3.8% among those aged 18–44 years to 10.7% among those aged ≥65 years. Among women, the percentage increased from 5.8% for those aged 18–44 years to 12.7% among those aged 45–64 years and 13.2% among those aged ≥65 years. Across all age groups, the percentage was higher among women than men.

**Source:** National Center for Health Statistics, National Health Interview Survey, 2017 and 2018. https://www.cdc.gov/nchs/nhis.htm.

